# Electrochemical fecal pellet sensor for simultaneous real-time *ex vivo* detection of colonic serotonin signalling and motility

**DOI:** 10.1038/srep23442

**Published:** 2016-03-22

**Authors:** Rachel Morris, Aidan Fagan-Murphy, Sarah J. MacEachern, Derek Covill, Bhavik Anil Patel

**Affiliations:** 1 Brighton and Sussex Medical School, Brighton, East Sussex, UK; 2 School of Pharmacy and Biomolecular Sciences, Brighton, East Sussex, UK; 3 School of Computing, Engineering and Mathematics, University of Brighton, Brighton, East Sussex, UK; 4 Hotchkiss Brain Institute, Calgary, Alberta, Canada; 5 Cumming School of Medicine, University of Calgary, Calgary, Alberta, Canada

## Abstract

Various investigations have focused on understanding the relationship between mucosal serotonin (5-HT) and colonic motility, however contradictory studies have questioned the importance of this intestinal transmitter. Here we described the fabrication and use of a fecal pellet electrochemical sensor that can be used to simultaneously detect the release of luminal 5-HT and colonic motility. Fecal pellet sensor devices were fabricated using carbon nanotube composite electrodes that were housed in 3D printed components in order to generate a device that had shape and size that mimicked a natural fecal pellet. Devices were fabricated where varying regions of the pellet contained the electrode. Devices showed that they were stable and sensitive for *ex vivo* detection of 5-HT, and no differences in the fecal pellet velocity was observed when compared to natural fecal pellets. The onset of mucosal 5-HT was observed prior to the movement of the fecal pellet. The release of mucosal 5-HT occurred oral to the fecal pellet and was linked to the contraction of the bowel wall that drove pellet propulsion. Taken, together these findings provide new insights into the role of mucosal 5-HT and suggest that the transmitter acts as a key initiator of fecal pellet propulsion.

Serotonin (5-HT) is present in enterochromaffin (EC) cells located in the colonic mucosal epithelium. As 90% of bodies 5-HT is intestinal derived, 5-HT has a wide systemic impact on various physiological processes such as bone development^1^ and thus monitoring levels *in vivo* can provide key insight. Following chemical and/or mechanical luminal stimulation of EC cells, 5-HT is released to activate 5-HT receptors on intrinsic primary afferent neurons (IPANs). In turn this communication drives neurogenic responses in enteric neurons to direct motility patterns^2^,^3^,^4^,^5^. Therefore alterations in mucosal 5-HT has been implicated to regulate colonic function. A series of human and animal studies have shown changes in motility and 5-HT signalling in ageing, obesity, and gastrointestinal diseases such as inflammatory bowel diseases; however such findings are merely correlative with current methodologies^6^,^7^,^8^. This has led to various studies which have investigated the role of 5-HT released from EC cells on colonic motility, however many of these studies have indicated that mucosal 5-HT play little or no role in pellet propulsion^9^,^10^,^11^,^12^. Therefore, the role of mucosal 5-HT in regulating colonic motility is poorly understood and thus the effects of alterations in mucosal 5-HT signalling on colonic motility are not fully known.

Limited knowledge of the regulation of colonic motility by mucosal 5-HT is due in part to limitations in the current methodologies. At present, experiments have been carried out predominately using flat tissue preparations where phasic motility patterns are monitored simultaneously with 5-HT signalling^13^,^14^. Other approaches have utilised pharmacological agents to alter mucosal 5-HT signalling, however this approach is non-specific and can also target enteric 5-HT signalling^15^. Finally tryptophan hydroxylase-1 (TpH1) knockout animals have been utilised as a model to reduce synthesis of mucosal 5-HT in order to understand the contribution that EC cell-derived 5-HT plays on motility; however these animals had a much reduced pellet transit and oversized fecal pellets^10^. Although all these studies have provided some physiological insight, they are unable to mimic a realistic *in situ* scenario. One study has using isolated guinea pig colon, investigated the relationship between peristalsis and 5-HT release, where a carbon fibre microelectrode was placed at the anal end of the colon to detect 5-HT release. This study indicated that EC cell derived 5-HT release does not initiate colonic peristalsis as 5-HT release was observed post colonic contractions. However within this study the sensor was ~2–3 mm away from front of the fecal pellet and therefore this may cause a delay in the response between the transducer and electrode^13^.

Here, we describe the fabrication and assessment of an electrochemical sensor intended to mimic a fecal pellet, which can be utilised to study fecal pellet propulsion. The device can be used to provide simultaneous detection of luminal 5-HT overflow from the mucosa while monitoring colonic motility of an intact *ex vivo* segment of colon via video imaging. Devices were developed that allowed for the means to monitor how mucosal 5-HT release varied at different regions of the fecal pellet. Studies were also conducted to investigate if 5-HT or fecal pellet propulsion occurred first and how the 5-HT selective reuptake inhibitor fluoxetine alters both 5-HT signalling and motility.

## Results

### Electrochemical characterisation of sensor fecal pellets

**Figure 1:**
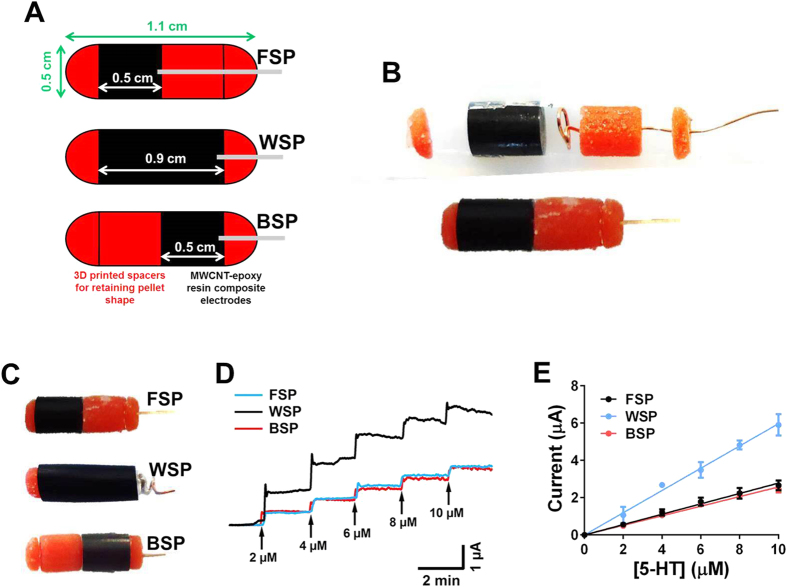
Fabrication and characterisation of the sensor pellet variations. **(A)** Schematic of the front (FSP), whole (WSP) and back (BSP) sensor pellet devices, where the black component is the sensor device and the red component is the 3D printed housing. All devices were 1.1 cm in length and 0.5 mm in diameter, which is similar in size to a natural fecal pellet. **(B)** Components showing how the devices were fabricated and electric connection were achieved in the final assembly. **(C)** Shows a photograph of the front (FSP), whole (WSP) and back (BSP) sensor pellet devices. **(D)** Amperometric assessment of the three pellet devices using fixed concentrations of 5-HT. Measurements were carried out in stirred solutions, where additions were made every 2 minutes and the change in current monitored. Electrochemical measurements were carried out at +600 mV vs Ag|AgCl reference electrode. **(E)** Calibration responses obtained from the various sensor pellet devices following amperometric assessment in varying concentrations of 5-HT (n = 10).

To investigate the ability of the devices to detect 5-HT, the front sensor pellet (FSP), whole sensor pellet (WSP) and back sensor pellet (BSP) devices were investigated using fixed concentrations of 5-HT. Figure 1D shows amperometry traces obtained on the various sensor pellet devices in stirred solutions, where increasing concentrations of 5-HT (from 2 μM to 10 μM) are added every 2 mins. These concentrations represent the range of values observed from other studies conducting electrochemical monitoring of mucosal 5-HT release^16^,^17^. Stable current responses are observed on the FSP, WSP and BSP devices. There was a slight drift in the signal on the WSP device. The devices are stable for 30 mins in concentrations of 10 μM, which is maximum time for a fecal pellet motility trial. The resultant calibration in Fig. 1E shows good linearity where the sensitivity for the FSP, WSP, and BSP devices were 278 ± 6 nM μA^−1^, 597 ± 10 nM μA^−1^ and 257 ± 4 nM μA^−1^ respectively. There was less than 5% variation between all FSP, WSP and BSP devices.

### *Ex vivo* characterisation of sensor pellet devices

**Figure 2:**
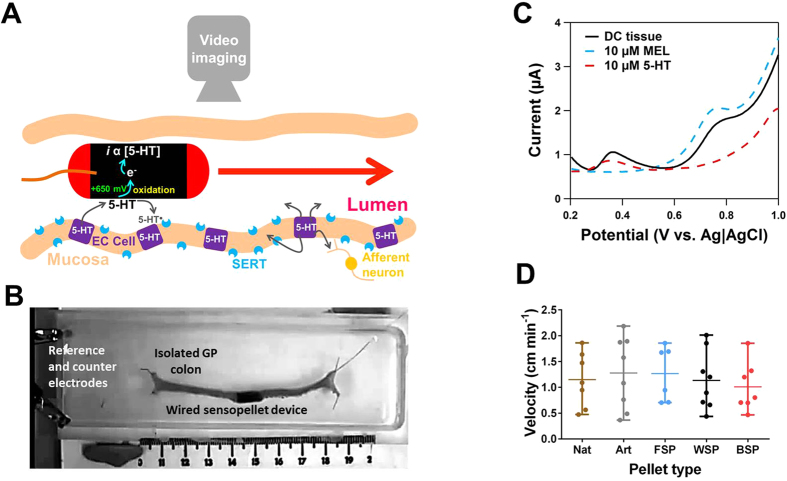
*In vitro* assessment of the various sensor pellet devices. **(A)** Schematic diagram showing how the sensor pellet device allows the simultaneous measurement of 5-HT overflow and motility. **(B)** Differential potential voltammogram using a WSP device *in vitro* in distal colon tissue. On comparison with fixed concentration standards of 5-HT and melatonin, the presence of both components were monitored in the distal colon lumen. **(C)** Video tracking of an *in vitro* recording of isolated distal colon tissue. The sensor pellet can be easily tracked via video imaging and the device through its wired connection allows for electrochemical detection of 5-HT**. (D)** Assessment of colonic motility of natural (nat), artificial (art, epoxy coated natural fecal pellet) and fabricated sensor pellets. No significant difference was between the *in vitro* colonic velocity of the natural fecal pellets and those of the wired sensor pellet devices (n = 6–8).

For biological recordings our device was inserted into the lumen of the proximal end of the isolated colon and the 5-HT oxidation current was monitored using amperometry, while video imaging was tracking the movement of the pellet. A schematic (Fig. 2A) and photograph (Fig. 2B) of the experimental set up demonstrates how the wired pellet sensor device is utilised for *ex vivo* measurements (see Supplementary Video). To understand if the device is able to detect mucosal release of 5-HT, differential pulse voltammograms were carried out whilst the WSP was positioned in the centre of the isolated colon. Figure 2C shows that current peaks were observed at +375 mV and +775 mV, which correspond with standards of 5-HT and melatonin, respectively. Similar responses were observed for the FSP and BSP. No differences in the ferricyanide response before and after *ex vivo* measurements on all devices was observed. The fecal pellet sensor mimics the shape and size of a natural fecal pellet in order to provide an appropriate stimulus to the intestinal tract in order to drive colonic motility. The velocities of natural and epoxy-coated artificial faecal pellets, which are conventionally used for motility assays^18^, were compared with the wired FSP, WSP and BSP. No significant difference in the velocity was observed indicating that the colons respond similarly to the sensor pellet devices when compared to the natural and artificial fecal pellets (Fig. 2D), thereby validating the suitability of the device for tracking signalling and motility simultaneously.

### Mucosal 5-HT is released at the back of the fecal pellet

**Figure 3:**
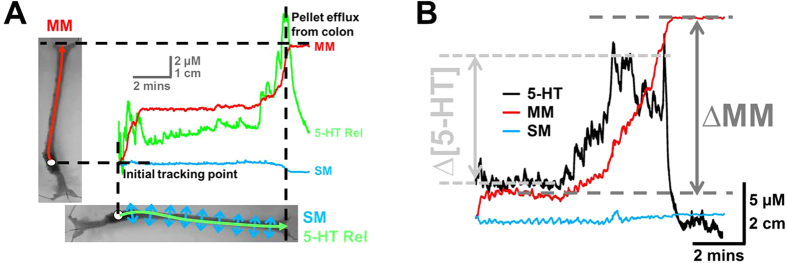
Interpretation of traces obtained using the sensor pellet devices *in vitro*. **(A)** The motility can be monitored using video imaging in the horizontal and vertical plane. Direct movement of the pellet from oral to anal is named motility movements (MM) and is governed by peristalsis. Lateral movements of the pellet were named sideways movements (SM). 5-HT release (5-HT rel) was monitored in real time during the course of a MM. All traces are plotted together with time being the x-axis so that correlations between 5-HTrel, MM and SM could be obtained**. (B)** Major changes in the amplitude of the responses were utilised for comparing the association between MM and 5-HT release. The major change from baseline of 5-HT release was recorded as ∆[5-HT], whist the major movement of the pellet from oral to anal was recorded at ∆MM.

**Figure 4:**
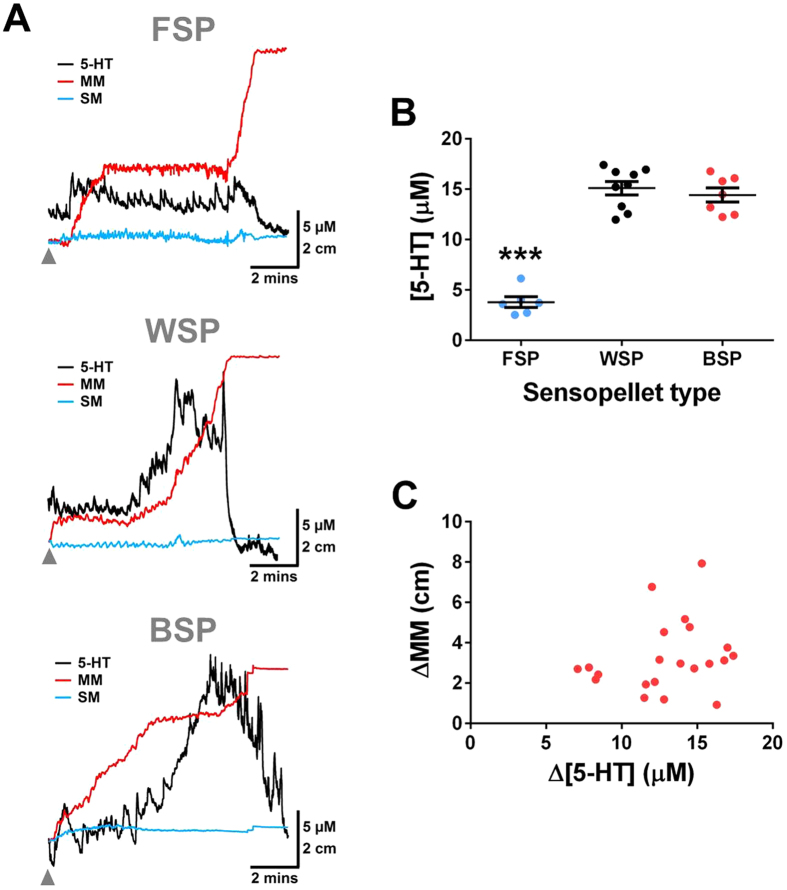
Understanding how mucosal 5-HT release alters at different locations of the fecal pellet **(A)** Amperometric and video image traces obtained in isolated colonic tissue segments using the FSP, WSP and BSP devices. The grey arrow on the traces indicates the point the device was inserted into the colon. **(B)** ∆[5-HT] was recorded on FSP, WSP and BSP devices, where a larger response was only noted on the WSP and BSP (n = 6–9). **(C)** Correlation of the ∆[5-HT] versus the ∆MM on the WSP and BSP. No direct correlation was observed indicating that the amount of 5-HT released is not directly correlated with the strength of the MM.

Although various studies have investigated the relationship between mucosal 5-HT release and motility patterns^14^,^19^, the majority of these have all been on flat tissue preparations^12^,^18^, and therefore little is known about how 5-HT release varies arounds a fecal pellet during motility. During recordings, luminal 5-HT overflow, motility movement that allows for propulsion of the fecal pellet (MM), and lateral movement of the colonic bowel wall (SM) can all be monitored simultaneously (Fig. 3A). Simultaneous recordings of MM, SM and mucosal 5-HT overflow were monitored on the FSP, WSP and BSP during colonic motility measurements. Representative traces are shown in Fig. 4A, where the MM resulted in the expulsion of the fecal pellet. Large increases in 5-HT overflow during the vicinity of the MM were only observed in the WSP and BSP. There was a significantly greater change in 5-HT concentration (∆[5-HT]) during the course of a MM (∆MM) in the WSP and BSP when compared to the FSP (Fig. 4B). This indicates that mucosal 5-HT is predominately released at the back of the fecal pellet. As observed in the Supplementary Video, relaxation of the bowel wall occurs oral to the fecal pellet prior to a contraction at the back of the fecal pellet to drive colonic motility. The ∆[5-HT] was compared to the ∆MM to understand if there was a correlation between the amount of 5-HT released to the strength of the MM. Figure 4C shows there is no correlation between these two parameters.

### Colonic mucosal 5-HT is released prior to motility movements that drive fecal pellet motility

**Figure 5:**
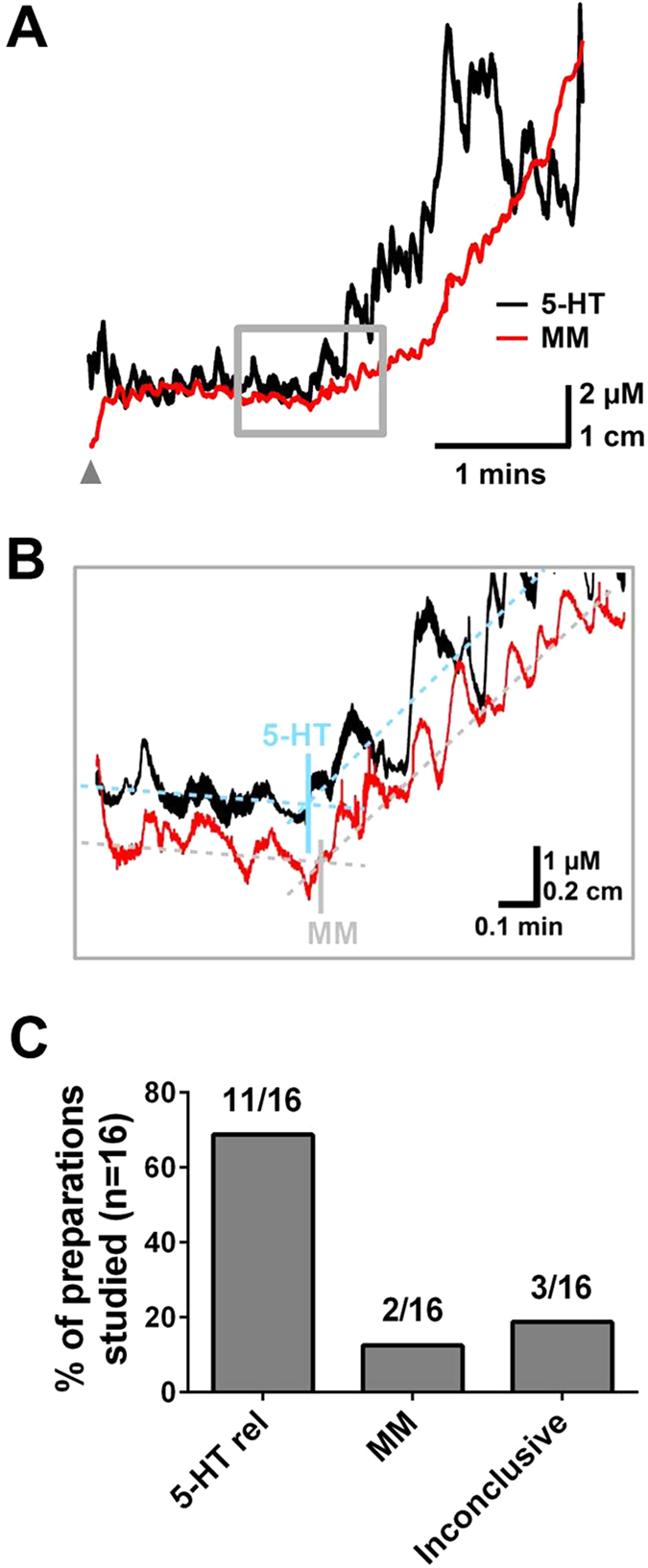
Understanding the association between mucosal released 5-HT and colonic motility. **(A)** shows a response from a WSP, where the box indicates the where a change in 5-HT release and pellet propulsion occurs. The grey arrow on the traces indicates the point the device was inserted into the colon. **(B)** The point of inflection between the basal response and rise in the signal was utilised to tract if the 5-HT release (5-HT rel) or the MM occurred first. **(C)** The number of preparations where 5-HT release or MM occurred first. The results indicate the 5-HT release occurs prior to the MM in the majority of preparation (n = 16).

There have been various studies that have called into question the role of mucosal 5-HT and the role it plays in driving motility^10^,^11^,^20^,^21^. In order to understand if MM or 5-HT overflow release occurred first, analysis of the inflection points in the 5-HT overflow and MM traces were carried out to understand which component initiates intestinal motility (Fig. 5A,B). In 11 out of 16 preparations 5-HT release preceded the MM, in 2 preparations the reverse was observed and in 3 preparations it was inconclusive to indicate which parameter proceeded (Fig. 5C). These findings clearly indicate that a change in luminal 5-HT usually occurs prior to a MM that drives fecal pellet propulsion.

### 5-HT selective reuptake inhibitor alters both mucosal 5-HT signalling and colonic motility

**Figure 6:**
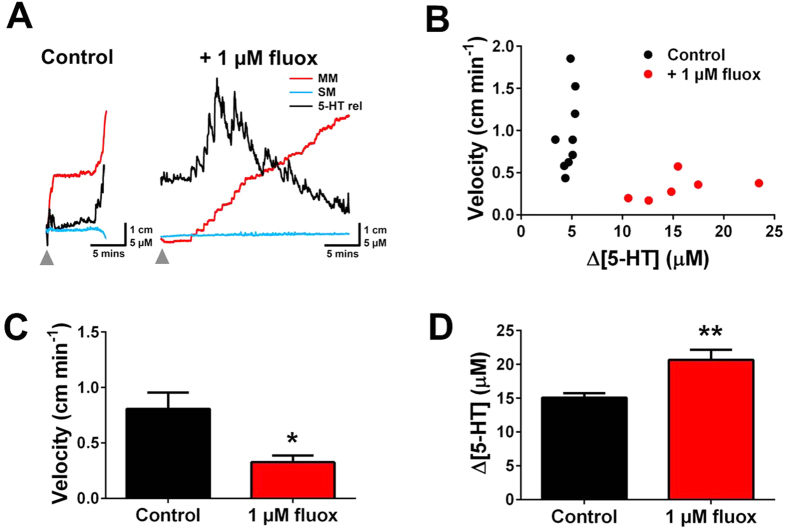
Influence of a serotonin selective reuptake inhibitor on 5-HT overflow and colonic motility. **(A)** Variations in MM, SM and 5-HT release in the presence and absence of 1 μM fluoxetine (1 μM fluox). There is a reduction in the rate of motility and an increase in the amount of 5-HT observed. The grey arrow on the traces indicates the point the device was inserted into the colon. **(B)** Correlation investigating the relationship between fecal pellet motility and ∆[5-HT] (n = 6–9). **(C)** A significant decrease in the velocity of the fecal pellet was observed in the presence of 1 μM fluoxetine (n = 6–9). **(D)** A significant increase in the ∆[5-HT] was observed in the presence of 1 μM fluoxetine (n = 6–9).

The influence of the 5-HT transporter (SERT) inhibitor, fluoxetine (1 μM) on motility and 5-HT overflow was investigated (Fig. 6). Previous studies have shown that this concentration of fluoxetine should reduce colonic motility and increase 5-HT overflow^18^. Traces in the presence of 1 μM fluoxetine show an increase in 5-HT overflow and an increase in the time taken for MM to complete when compared to control (Fig. 6A). As shown in Fig. 6B, there is a significant increase in 5-HT overflow that results in a decrease in fecal pellet velocity. There is a significant decrease in the velocity of the fecal pellet sensor in 1 μM fluoxetine (Fig. 6C). A significant increase in the ∆[5-HT] was observed in the presence of 1 μM fluoxetine (Fig. 6D). An inhibition of SERT leads to increased observed mucosal 5-HT overflow, which could cause desensitisation of 5-HT receptors in the IPANs, leading to reduced motility. The device is capable of tracking these events by monitoring both signalling and motility.

## Discussion

Mucosal 5-HT is an important neurochemical within the colon and is present in EC cells, where it has been shown to be influence motility. However in recent years there has been much debate into the role 5-HT plays in motility patterns and if it has a function at all^21^. Such confusion on the role of mucosal 5-HT is due to various studies that have shown contradictory views on its importance in generation of colonic migratory motor complexes (CMMCs) in mice^9^,^11^,^22^. However all these studies have not simultaneously evaluated mucosal release and colonic motility in timescales where their relationship can be fully understood. This has been mainly due to the difficulties in development of a methodology that allow for tracking of the release profile of 5-HT in the lumen simultaneously with the colonic motility patterns.

The pellet sensor device developed within this study mimics the shape and size of a natural fecal pellet and therefore provides a suitable means of monitoring 5-HT release within the direct vicinity of the fecal pellet during propulsion. The fecal pellet sensor also cause distension to the colon wall and induces a realistic mechanical stimulus to the mucosal epithelium to drive 5-HT release. The fabrication of the sensor pellet device itself is also very flexible, as the electrode position, shape, size and length of the device can be varied. Therefore although the device has been studied in the guinea pig colon within this study, there is scope for it to be used in other mammalian systems.

Characterisation of the various fecal pellet sensor devices showed that the devices were suitable for stable and sensitive detection of 5-HT. We have previously shown that compressed MWCNT composite electrodes are less prone to fouling and show good stability for detection of 5-HT^23^, which makes them attractive for luminal monitoring. When sensor pellet devices were placed within the *ex vivo* colon, current responses were observed, which corresponded to 5-HT and melatonin standards. Various electrochemical studies have also monitored luminal 5-HT and melatonin^8^,^16^,^24^ These findings indicate that a fraction of the transmitter is released into the lumen, as the majority of EC cell derived 5-HT is released basolateral to stimulate 5-HT receptors on IPANs. It is not fully understood, why luminal release of 5-HT and melatonin is observed, however this could be due to leakage through tight junction or may be a communication to the gut microbiota^25^. The concentration of 5-HT observed in our study was within the range observed using sensor studies on *in vitro* tissue from various mammalian species^17^.

The major concern with a sensor pellet device is that the device itself alters normal physiological behaviour and therefore studies were conducted to investigate if fecal pellet propulsion was altered using the developed device, in comparison to that of an artificial and natural fecal pellet. Fecal pellet motility assays are very common, and often are conducted using an epoxy-coated fecal pellet in order to conduct multiple trials and investigate the physiology and pharmacology of pellet motility^18^. All our sensor pellet devices showed similar velocity and movement patterns in the colon when compared to the natural and commonly used artificial fecal pellets. These findings suggest that the wired device does not influence or alter the normal behaviour of the *ex vivo* tissue.

Using our fecal pellet devices, we investigated the temporal dynamics between the onset of a major motility movement and changes in mucosal 5-HT current signal. In the majority of preparations we observed that onset of mucosal 5-HT release occurred initially, prior to the motility movement. This would infer that mucosal 5-HT release was an important part of the process that initiates the movement of a fecal pellet. Another study conducted in mice, looked at the association of 5-HT and CMMCs, have indicated that localised release of 5-HT at the pellet may be important in this specific motility pattern^9^, however others have refuted this notion^11^. In guinea pigs, studies have focused on how distension-evoked peristalsis is influenced by mucosal 5-HT, where finding indicate that there is no specific role^13^. When distension-evoked peristalsis was investigated, a fixed fecal pellet was placed in the isolated colon and a carbon fibre microelectrode was placed within ~2–3 mm of the fecal pellet to record responses. The distance between the electrode and pellet may induce a delay in the temporal dynamics of the two responses, which may explain the difference between their study and ours. Although within our study we do not indicate if mucosal 5-HT is needed at all for distension-evoked peristalsis, our finding strongly suggests a relationship between EC cell 5-HT and motility. Therefore we feel that mucosal 5-HT could either be acting as an initiator of colonic motility patterns that help to govern fecal pellet propulsion.

To further support this notion, we have the ability to tailor the fecal pellet sensor so that specific regions of the fecal pellet can be utilised for sensing luminal 5-HT, we were able to understand if the release of 5-HT from the EC cells was either oral or anal to the fecal pellet. Following assessment of the video recordings and the 5-HT response, during a fecal pellet movement, we initially observe a relaxation anal to the fecal pellet; this is then followed by release of luminal 5-HT on the oral side of the fecal pellet, that is then closely followed by a contraction that propelled the fecal pellet down the colon. This result suggests that 5-HT release from mucosal EC cells drives the enteric circuitry associated with colonic contraction during fecal pellet propulsion. This provides useful information into the role of mucosal signalling and that specifically of 5-HT, as many studies have shown that exogenous application of 5-HT to colonic tissue will induce a biphasic response^26^,^27^. This biphasic response may be due to the actions of mucosal 5-HT on directing contraction through enteric neurons, whilst relaxation by be induced through enteric 5-HT release on enteric neurons. Our finding also showed that the amount of 5-HT release did not govern the strength of contraction or degree of which the fecal pellet was propelled down the colon. This also supports our notion that 5-HT release from the mucosa initiates fecal pellet propulsion.

Our device was also altered in the presence of SERT inhibitor fluoxetine, which at concentrations utilised has been shown to reduce colonic motility^28^ and increase overflow of mucosal 5-HT^29^. Our device showed that in the presence of fluoxetine there was a significant reduction in the fecal pellet propulsion velocity as well as a significant increase in the 5-HT overflow. Not only does these findings validate that our device is a suitable means of simultaneously tracking motility and signalling, but also it showcases the potential of the device for conduct preclinical evaluation of new lead compounds for intestinal disorders.

Finally, although a powerful approach, the fecal pellet device used here is not without limitations. Although the device provides real-time data on motility and signalling, it cannot provide in-depth mechanistic information and a true reflection of *in vivo* behaviour and therefore this need to be taken into consideration when interpreting the data. The wider impact of the fecal sensor pellet will be based on conducting measurement *in vivo* and understanding if changes in signalling can explain impairment in colonic motility in ageing, obesity and intestinal disease.

## Methods

### Fabrication of the sensor pellet device

Each sensor pellet model consists of a single carbon-composite electrode produced by compression^23^ and two or more non-conducting sections produced by 3D printing (Fig. 1). For the fabrication of the conductive carbon-composite electrode, 25% of 30–50 nm multiwall carbon nanotube (MWCNT; Cheaptubes, USA) was mixed with 75% of epoxy resin (3 part epoxy resin to 1 part hardener; Robnor Resins Ltd, UK) to generate a paste, which was packed into 3.2 mm tubing and between 300–320 psi was applied to compress the composite. Following curing for 48 hours at room temperature, the electrodes were cut using diamond wafer blade (Buehler saw) to the lengths indicated in Fig. 1A. Fabrication of the components that were utilised to house the electrode and provide the shape features of the fecal pellet were designed using Solidworks (Dassault Systèmes Inc), exported as stereolithography files, and were printed in ABS using a Replicator 2X experimental 3D printer (Makerbot Inc). Parts were specifically designed to require no support material to make post processing as straight forward as possible. Conductive and non-conductive parts of the device were connected using a thin layer of adhesive glue (Fig. 1B). Electrical contact was achieved by connecting the carbon-composite electrode to insulated copper wire using silver-loaded epoxy resin (Circuit Works, RS Components, Corby, UK). Three different manifolds were created: one where the whole linear portion of the device was electroactive (WSP), one where the first 5 mm of the device was able to sense (FSP) and finally where the final 5 mm of the device was able to sense (BSP, Fig. 1C). In order to assure the outside surface of the device shape has a uniform surface, the whole device was sequentially polished with 1.0 μm, 0.3 μm and 0.05 μm alumina powder.

### Sensitivity and stability of sensor pellet devices

To monitor the sensitivity of the FSP, WSP and BSP, calibration responses were obtained in 5-HT solutions ranging from 2 to 10 μM. Devices were placed in stirred solutions at 20 rpm and exposed to increasing concentrations of 5-HT every 2 minutes. Amperometry detection was carried out at +600 mV vs Ag|AgCl reference electrode.

To monitor the *ex vivo* stability of the devices, each device was placed in 1 mM potassium ferricyanide prior to biological measurements and cyclic voltammetry was run between −100 mV to +600 mV at a 100 mV s^−1^ scan rate. This response to 1 mM potassium ferricyanide was then repeated following a biological recording and the change in the current from the initial measurement was monitored on the FSP, WSP and BSP devices.

### Animals

All procedures were carried out according to U.K. Home Office regulations and were approved by the University of Brighton Ethics Committee. Male Duncan Hartley guinea-pigs weighing 300–400 g were euthanized using CO_2_ followed by severing of the carotid arteries. A 5 cm segment of colon was harvested 5 cm from the anus and placed in oxygenated (95% O_2_ and 5% CO_2_) Kreb’s buffer solution pH 7.4 (117 mM NaCl, 4.7 mM KCl, 2.5 mM CaCl_2_, 1.2 mM MgCl_2_, 1.2 mM NaH_2_PO_4_, 25 mM NaHCO_3_, and 11 mM glucose) at 37 °C prior to experiments.

### Simultaneous monitoring of 5-HT overflow and motility using video recordings

The *ex vivo* 5 cm segment of the colon was placed in a sylgard lined flow bath and filled with Kreb’s buffer (37 °C). A small 2 mm incision was made at both the distal and proximal end of the colon and pins were placed in both the openings of the colon segment in order to lightly stretch and hold it in place. Colon preparations were left for 30 minutes in order for the natural expulsion of fecal matter to occur. During this time the velocity of these natural pellets was recorded by recording colon segments using a video camera, and tracking pellet movement through the colon using EthoVision XT video tracking software (Noldus). Any faecal matter remaining after thirty minutes was flushed through the colon segment using a syringe of warm Kreb’s solution. Prior to using the sensor pellet device, a recording of fecal pellet motility was conducted using an epoxy-coated faecal pellet.

For monitoring 5-HT overflow, constant-potential amperometry was utilised. A stainless steel rod and non-leak Ag|AgCl reference electrode (Cyprus Systems, USA) were placed in the flow bath. The sensor pellet device served as the working electrode and was held at +600 mV vs Ag|AgCl using a CHI1206B potentiostat (CH Instruments, USA). During this period the tracking of the pellet movement was carried out simultaneously. All recordings were terminated upon pellet expulsion or at thirty minutes. Two trials were carried out on each of the FSP, WSP, BSP. The effect of 1 μM fluoxetine (Sigma-Aldrich, USA) on 5-HT release and motility was also investigated.

### Data analysis

Not all pellets propagated through the full length of the colon segments, however many of these still demonstrated major propagation movements (periods of sustained movement ≥3 cm). It was decided that only recordings where the pellets travelled ≥3 cm would be accepted for further analysis. Movements in the X (motility movements, MM) and Y (sideways movements, SM) planes were obtained from the video tracking software. Data from the electrochemical software and the video tracking software was compiled and were graphically represented using Igor Pro 4.03 for further analysis. Recordings were represented graphically by plotting both current and movement as a function of time (Fig. 3A).

Pellet velocity was calculated as the total distance moved by the pellet, either in the time to expulsion or in thirty minutes if pellets were not expelled. On all traces, the major propagation movements were monitored. The change in motility movement (∆MM) and the change in 5-HT concentration response (Δ[5-HT]) were recorded for each major propagation movement on the WFS, FSP and BSP device traces (Fig. 3B).

In order to evaluate the temporal relationship between 5-HT release and motility movements, the onset of the motility movement and the associated 5-HT current response were analysed. To determine the time of onset, a line of best fit was calculated for the basal level of 5-HT, and another line of best fit was calculated for the large increase in release. The point of intersection was found and the time was recorded as the onset of the 5-HT peak. The same was done for the MM trace to determine the onset time of the major propagation movement. The onset times were then compared to see whether 5-HT release or MM occurred first.

All data was plotted as mean ± standard deviation using GraphPad Prism. Data were statistically assessed using one-way and two-way ANOVAs with Bonferroni post-tests. P < 0.05 was considered as statistically significant.

## Additional Information

**How to cite this article**: Morris, R. *et al*. Electrochemical fecal pellet sensor for simultaneous real-time *ex vivo* detection of colonic serotonin signalling and motility. *Sci. Rep*. **6**, 23442; doi: 10.1038/srep23442 (2016).

**Data Availability**: Data associated with this study can be found from the DOI: 10.17033/ DATA.00000011.

## Supplementary Material

Supplementary Information

Supplementary Video S1
