# Supplementary material for: Electrochemical fecal pellet sensor for simultaneous real-time ex vivo detection of colonic serotonin signalling and motility
Source: Sci Rep. 2016 Mar 22;6:23442. doi: 10.1038/srep23442 (PMC4802304; doi:10.1038/srep23442)
Supplement: Supplementary Information [file srep23442-s1.doc]

**Electrochemical fecal pellet sensor for simultaneous real-time *ex vivo* detection of colonic serotonin signalling and motility**

Rachel Morris^1^, Aidan Fagan-Murphy^2^, Sarah J. MacEachern^4,5^, Derek Covill^3^ & Bhavik Anil Patel^2,*^

**Supplementary Video**

Video showing how the fecal pellet device can be utilised to track fecal pellet motility and serotonin signalling simultaneously. The wired fecal pellet device was inserted approximately 0.5 cm into the oral end of the distal colon. The pellet device was allowed migrate down the colon naturally in warm (37°C) oxygenated Krebs buffer solution. The reference and counter electrodes were placed in the right hand side of the flow bath. Video tracking was utilised to study the fecal pellet motility, whilst the current observed due to the oxidation of serotonin was monitored using a potentiostat.
